# Overexpression of UBE2C in esophageal squamous cell carcinoma tissues and molecular analysis

**DOI:** 10.1186/s12885-021-08634-6

**Published:** 2021-09-06

**Authors:** Rong Li, Xing-Feng Pang, Zhi-Guang Huang, Li-Hua Yang, Zhi-Gang Peng, Jie Ma, Rong-Quan He

**Affiliations:** 1 Department of Medical Oncology, The First Affiliated Hospital of Guangxi Medical University, Nanning, China; 2 Department of Pathology, The First Affiliated Hospital of Guangxi Medical University, Nanning, China

**Keywords:** Ubiquitin conjugating enzyme E2 C, Esophageal squamous cell carcinoma, RNA-sequencing, Tissue microarray, Immunohistochemistry

## Abstract

**Background:**

Esophageal cancer is a common malignant tumor and its 5-year survival rate is much lower than 30% due to its invasiveness and pronounced metastasis ability, as well as the difficulty in early diagnosis. This study aimed to elucidate the molecular mechanism of ubiquitin conjugating enzyme E2 C (UBE2C) in esophageal squamous cell carcinoma (ESCC).

**Methods:**

In this study, we conducted a comprehensive evaluation of the UBE2C expression in ESCC by collecting the protein and mRNA expression data (including in-house RNA-seq, in-hosue immunohistochemistry, TCGA-GTEx RNA-seq and tissue microarray) to calculate a combined standardized mean difference (SMD) and summary receiver operating characteristic curve (sROC). Kaplan-Meier (K-M) method was used for survival analysis. We also explored the mechanism of UBE2C in ESCC by combing the differentially expressed genes (DEGs) of ESCC, related-genes of UBE2C in ESCC and the putative miRNAs and lncRNAs which may regulate UBE2C.

**Results:**

UBE2C protein and mRNA were highly expressed in ESCC tissues (including 772 ESCC tissue samples and 1837 non-cancerous tissue control samples). The pooled SMD of UBE2C expression values was 1.98 (95% CI: 1.51–2.45, *p* < 0.001), and the the area under the curve (AUC) of the sROC was 0.93 (95% CI: 0.90–0.95). The results of survival analysis suggested that UBE2C is likely to play different roles in different stages of the ESCC. Pathway anaylsis showed that UBE2C mainly influenced the biological function of esophageal cancer by synergistic effects with CDK1, PTTG1 and SKP2. We also constructed a potential UBE2C-related ceRNA network for ESCC (HCP5/has-miR-139-5p/UBE2C).

**Conclusion:**

UBE2C mRNA and protein level were highly expressed in ESCC and UBE2C was likely to play different roles in different stages of the ESCC.

**Supplementary Information:**

The online version contains supplementary material available at 10.1186/s12885-021-08634-6.

## Introduction

Esophageal cancer is a common malignant tumor that originates in the esophageal epithelium or glands with a 5-year survival rate less than 30%. China has one of the highest incidence rates and mortality rates for esophageal cancer, mainly in the form of esophageal squamous cell carcinoma (ESCC) [1, 2]. Therefore, more research is needed on the molecular mechanism of esophageal cancer to identify effective molecular targets and to screen the population likely to benefit from that targeting.

One of the numerous potential molecular mechanisms underlying esophageal cancer may involve ubiquitin conjugating enzyme E2 C (UBE2C), a protein-coding gene related to pathways such as the cell cycle and mitosis that modulate the progression of cancer [3]. UBE2C overexpression has been confirmed in many kinds of cancer tissues [4]. However, few studies have considered a potential role for gene regulation of UBE2C in ESCC.

In recent years, the discovery of the competitive endogenous RNA (ceRNA) mechanism has since attracted the attention of many researchers and has stimulated many relevant studies since Salmena et al. first proposed it [5]. In the tumor field, ceRNAs can be used as decoys to attract and isolate miRNAs, thereby relieving the inhibition of miRNA target genes and regulating the molecular biology and development of the tumor [6–8]. No association has yet been established between UBE2C expression and specific miRNAs and lncRNAs in ESCC.

In this study, we first performed in-house RNA-seq to determine the expression of UBE2C in ESCC tissues. Then immunohistochemistry (IHC) was used to verify the expression of this gene. Finally, we mined the public databases and conduct a comprehensive analysis of UBE2C expression based on in-house RNA-seq, in-house IHC and public databases. An in silico method was used to predict the upstream miRNAs and lncRNAs of UBE2C to elucidate the molecular mechanism of UBE2C in ESCC. This study can provide new ideas for the early diagnosis and clinical treatment of ESCC.

## Materials and methods

### In-house RNA-seq

ESCC and control tissues were collected from 8 patients with ESCC admitted to the First Affiliated Hospital of GuangXi Medical University (China). Total RNA (including mRNA and miRNA) was extracted from the collected tissues, and the expression value data of RNA were processed into the Transcripts Per Million (TPM) and presented in log2 form. This study was approved by the ethics committee of the First Affiliated Hospital of Guangxi Medical University in accordance with the Declaration of Helsinki, and each patient provided written consent to participate.

### Immunohistochemistry

The normal formalin fixed and paraffin-embedded (FFPE) tissues of 162 cases of ESCC and of 162 paired controls were obtained from Fanpu Biotech, Inc. This study was approved by the ethics committee of the First Affiliated Hospital of Guangxi Medical University, and the written consent was obtained from each patient. Each case was evaluated in a double-blinded manner by two experienced pathologists. If the two pathologists’ interpretations were inconsistent, the case was further reviewed by a third pathologist. The positive controls were required to show positive results, and the negative controls were required to show negative result on each test. The staining intensity was evaluated by light microscopy using a four-layer grading system of “no staining,” “weak staining,” “medium staining,” and “strong staining,” corresponding to 0, 1, 2, and 3 points. At the same time, the cell positive rate was scored according to the following scoring criteria: 0 points = no cell staining; 1 point =0–25% cells stained; 2 points = 26–50% cells stained; 3 points = 51–75% cells stained; 4 points = over 76% cells stained. An immunoreactive scoring system of 0–12 points was constructed according to the intensity and positive rate of cell staining determined by light microscopy. The relationship between UBE2C expression and the pathological parameters was then analyzed.

### RNA-seq and tissue microarray date in the universal database

The results from the in-house ESCC tissue RNA-seq and IHC were validated by downloading the esophageal cancer dataset in the Cancer Genome Atlas (TCGA) database from the UCSC Xena browser (https://xena.ucsc.edu/), including the expression data for mRNA (including lncRNA) and miRNA, as well as the clinical follow-up information. We then discarded the samples of esophageal adenocarcinoma, leaving only ESCC. To make the results more reliable, we also included the expression data for normal esophageal tissues in the Genotype-Tissue Expression (GTEx) database for further analysis.

**Fig. 1:**
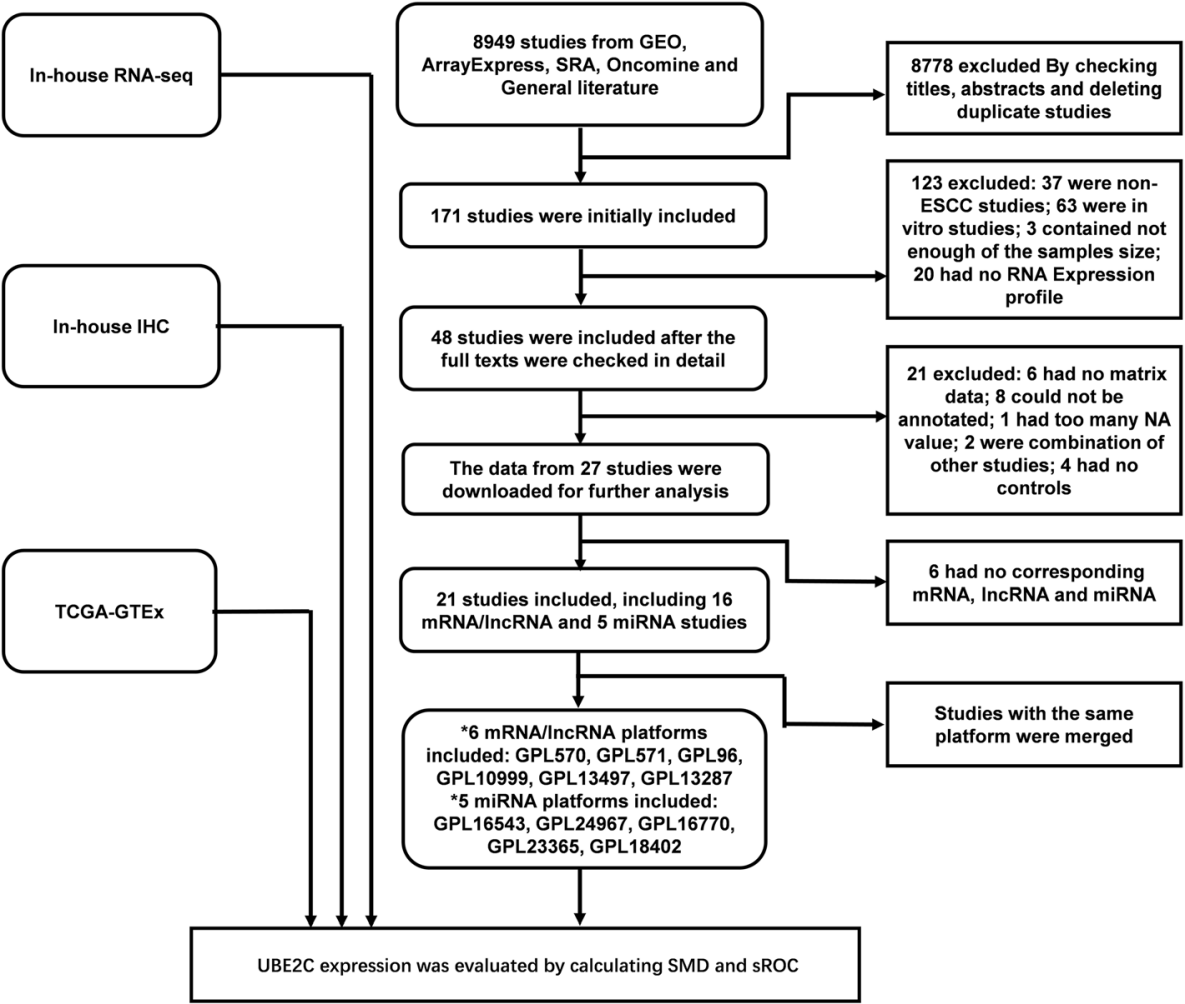
The flow chart of included studies *GPL570 (merging by GSE45670, GSE77861, GSE26886, GSE69925, GSE100942, GSE33810, GSE17351); GPL571 (merging by GSE20347, GSE38129, GSE29001, GSE33426); GPL96 (GSE23400); GPL10999 (GSE32424); GPL13497 (GSE45168); GPL13287 (GSE70409) *GPL16543 (GSE43732); GPL24967 (GSE114110), GPL16770 (GSE59973), GPL23365 (GSE112840), GPL18402 (GSE71043)

We further expanded the sample size by obtaining the RNA-seq and tissue microarray data for all mRNA, miRNA, and lncRNA expressions in human ESCC tissues and normal controls from the GEO, ArrayExpress, SRA, and Oncomine databases and available literature. The controls included tissues adjacent to ESCC, normal esophageal tissue, or humoral tissue from ESCC patients or healthy humans. The included tissue microarrays and RNA-seqs were preprocessed, and the tissue microarrays of the same platform were merged and batch processed. The search keywords were “Esophageal cancer.” The following were the inclusion criteria: the expression data (including the related mRNA, miRNA, or lncRNA) and their annotation information from RNA-seq and tissue microarrays must be available; the RNA-seq and tissue microarrays must contain both ESCC tissue and control tissues (adjacent tissue or healthy esophageal tissue) or include the humoral tissues of patients with ESCC and the humoral tissues of healthy people; the number of included samples in both experimental and the control groups must be 3 or more, or the total number of the combined experimental groups and the control groups must be 3 or more; and the RNA-seq and tissue microarrays must be based on human samples, not other animals. (Fig. 1). All the searching and inclusion work was completed by two people independently.

### The mRNA expression of UBE2C in ESCC cell lines

We downloaded the expression profile data for UBE2C in esophageal cancer cell lines from the CCLE (the Cancer Cell Line Encyclopedia), and screened the expression profiles of 27 esophageal cancer cell lines for further analysis.

### Copy number analysis of UBE2C

The underlying mechanism of the UBE2C gene mutation is still unclear. Therefore, this study used the cBioPortal database to perform copy number analysis for UBE2C in the esophageal cancer dataset [9, 10]. Gene copy number variation (CNV) detection is a method for detection of the variations in large DNA sequences in the genome by scanning a specific gene or the whole genome. The cBioPortal provides a visualization tool for research and analysis of cancer gene data to aid in understanding the molecular data obtained from cancer tissue and cell studies. In the present study, mutation data were selected from total exome sequencing from 559 patients with esophageal cancer from TCGA. The copy number variation of UBE2C was then automatically analyzed and the results were displayed directly on the web page.

### Enrichment analysis

We identified the differentially expressed genes (DEGs) between the cancerous tissue and the non-cancerous tissue in all the ESCC microarray data (including mRNA, miRNA and lncRNA) using the limma package of R software, and the DEGs in the TCGA-GTEx combined RNA-seq and the in-house RNA-seq using the R software package edgeR and DESeq2. The cut-off value was set to |log2FC| > 1, FDR < 0.05. Robust rank aggregation (RRA) and artificial ranking was used to select DEGs that appeared in at least two data sets as meaningful DEGs. The relevant genes for UBE2C in ESCC tissues were calculated using the Pearson correlation coefficient and the relevant genes appearing in at least three data sets were selected as the relevant genes of significance. The thresholds were set as |Pearson’s r| ≥ 0.5 and *p* < 0.05. The selected DEGs and related genes were plotted on a Venn map, and the genes at the intersection of the two were analyzed using the clusterprofiler package of R software for Gene ontology (GO) and Kyoto Encyclopedia of Genes and Genomes (KEGG) analysis. The Protein-Protein Interaction (PPI) network was mapped using the pathway genes of the first five pathways (with the lowest *p* values) in KEGG and visualized by metascape combined with cytoscape software [11].

### Prediction and validation of the ceRNA network

In this study, candidate miRNAs and lncRNAs were screened by combining online prediction databases and ESCC RNA-seq and tissue microarray data. We used 12 miRNA prediction tools (miRWalk, miRanda, miRDB, miRNAMap, PITA, RNAhybrid, Microt4, mirbridge, miRMap, Pictar2, RNA22, Targetscan) to predict upstream miRNAs of UBE2C. The predicted miRNAs and the differential expressed miRNAs that appeared in ESCC tissues in at least 2 datasets were selected for intersection, and the intersecting miRNAs was selected as candidate miRNAs. These candidate miRNAs were then used to predict upstream lncRNAs through the miRNet database. We then selected the intersection of predicted lncRNA and differentially expressed lncRNA in ESCC as candidate lncRNAs. Finally, cytoscape software was used to visualize the ceRNA network. The binding sites of miRNAs to UBE2C and of miRNA to lncRNA were obtained from the RNAhybrid database and starbase database, respectively.

### Statistical analysis

SPSS 17.0 software was used for all statistical analyses. Student’s t test was used to analyze the UBE2C protein, UBE2C mRNA, candidate miRNAs and candidate lncRNAs expression values in ESCC tissues and non-cancer tissues, as well as correlations with various clinical parameters. We used the receiver operating characteristic curve (ROC) and the area under the curve (AUC) to assess the ability of UBE2C to distinguish ESCC from non-cancerous tissue. (Note: the AUC ranges between 0.5 and 1. An AUC closer to 1.0 indicates a higher authenticity of the detection method. An AUC between 0.5 and 0.7 indicates a low diagnostic accuracy. An AUC between 0.7 and 0.9 indicates a certain accuracy of the diagnosis and an AUC greater than 0.9 indicates a higher accuracy. An AUC of 0.5 indicates the lowest authenticity and has no application value.) GraphPad Prism 8.0 software was used to draw violin diagrams and ROC curves.

Comprehensive and systematic analysis of UBE2C, candidate miRNAs and candidate lncRNAs expression was conducted using Stata 15.0 software. This study integrated the tissue microarrays and RNA-seq data to carry out the comprehensive analysis, using the standardized mean difference (SMD) and 95% confidence interval (CI) to further compare the UBE2C, candidate miRNAs and candidate lncRNAs expression in ESCC tissues and non-cancerous tissue. A total SMD > 0 and 95%CI  > 0 indicated upregulation of UBE2C, candidate miRNAs and candidate lncRNAs expression in ESCC compared with that in non-cancerous tissue. The specificity, sensitivity, negative likelihood ratio, and positive likelihood ratio were analyzed with the random effects model. We integrated all the data to draw the sROC curve for further clarification of the clinical diagnostic value of UBE2C for ESCC. The heterogeneity was expressed by Student’s t test. Values of *p* < 0.05 or I^2^ > 50% suggested that the results were heterogeneous and that a random effects model was warranted. Values of *p* > 0.05 or I^2^ < 50% indicated no significant heterogeneity of the results so a fixed effect model was used. Values of *p* < 0.05 were considered statistically significant.

GraphPad Prism 8.0 software was used to calculate the Pearson correlation coefficient for the ESCC dataset in the TCGA database and in house RNA-seq to verify the correlations associated with lncRNA, miRNA, and mRNA. A value of the correlation coefficient r between 0 and 1, with a value of *p* < 0.05, was considered to indicate a statistically significant positive correlation; a value of the correlation coefficient r between 0 and − 1, with a value of *p <* 0.05, was considered to indicate a statistically significant negative correlation. A value of *r =* 0 was considered to indicate no correlation.

### Survival analysis

The prognostic value of UBE2C in ESCC was analyzed using the Kaplan-Meier (KM)-plotter database. The KM-plotter can evaluate the effects of 54 K gene on the survival rate in more than 20 cancer types. The system includes microarray and RNA-seq data sources from the GEO and TCGA databases. In this study, ESCC mRNA RNA-seq data from the pan-carcinoma project in the KM-plotter database were selected. The 95% confidence interval risk and logrank *p* values of hazard ratio (HR) were then calculated automatically and displayed directly on the web page. Values of the logrank *p* < 0.05 were considered statistically significant [12].

## Results

### UBE2C mRNA levels indicated overexpression in in-house RNA-seq

**Fig. 2:**
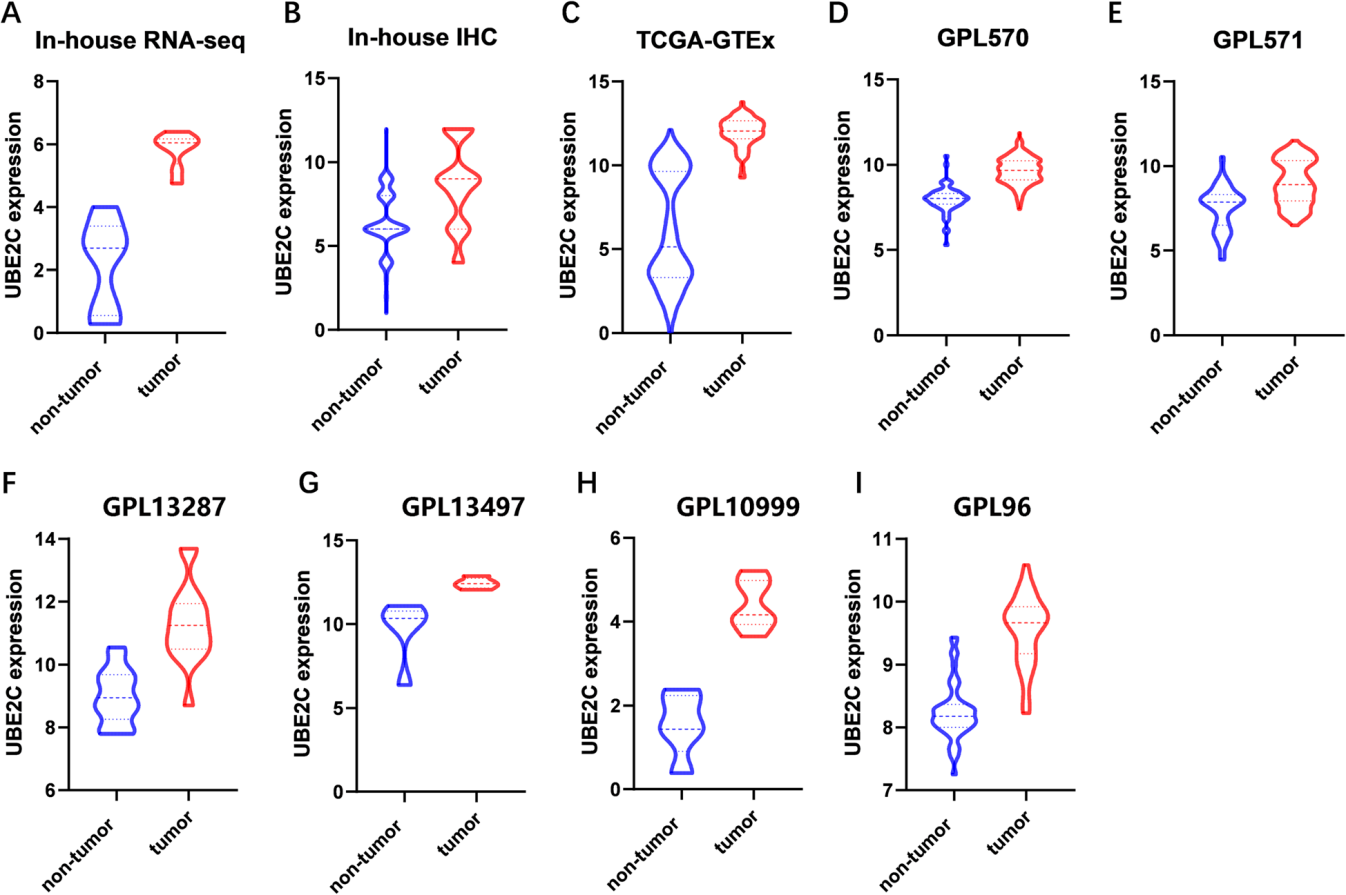
Violin plot of UBE2C mRNA and protein overexpression in ESCC tissues assessed by in-house RNA-seq, in-house IHC, TCGA-GTEx RNA-seq and tissue microarray. a, Violin plot of UBE2C mRNA based on in-house RNA-seq; b, Violin plot of UBE2C protein based on in-house IHC; c, Violin plot of UBE2C mRNA based on TCGA-GTEx RNA-seq; d-I, Violin plot of UBE2C mRNA based on tissue microarray

**Fig. 3:**
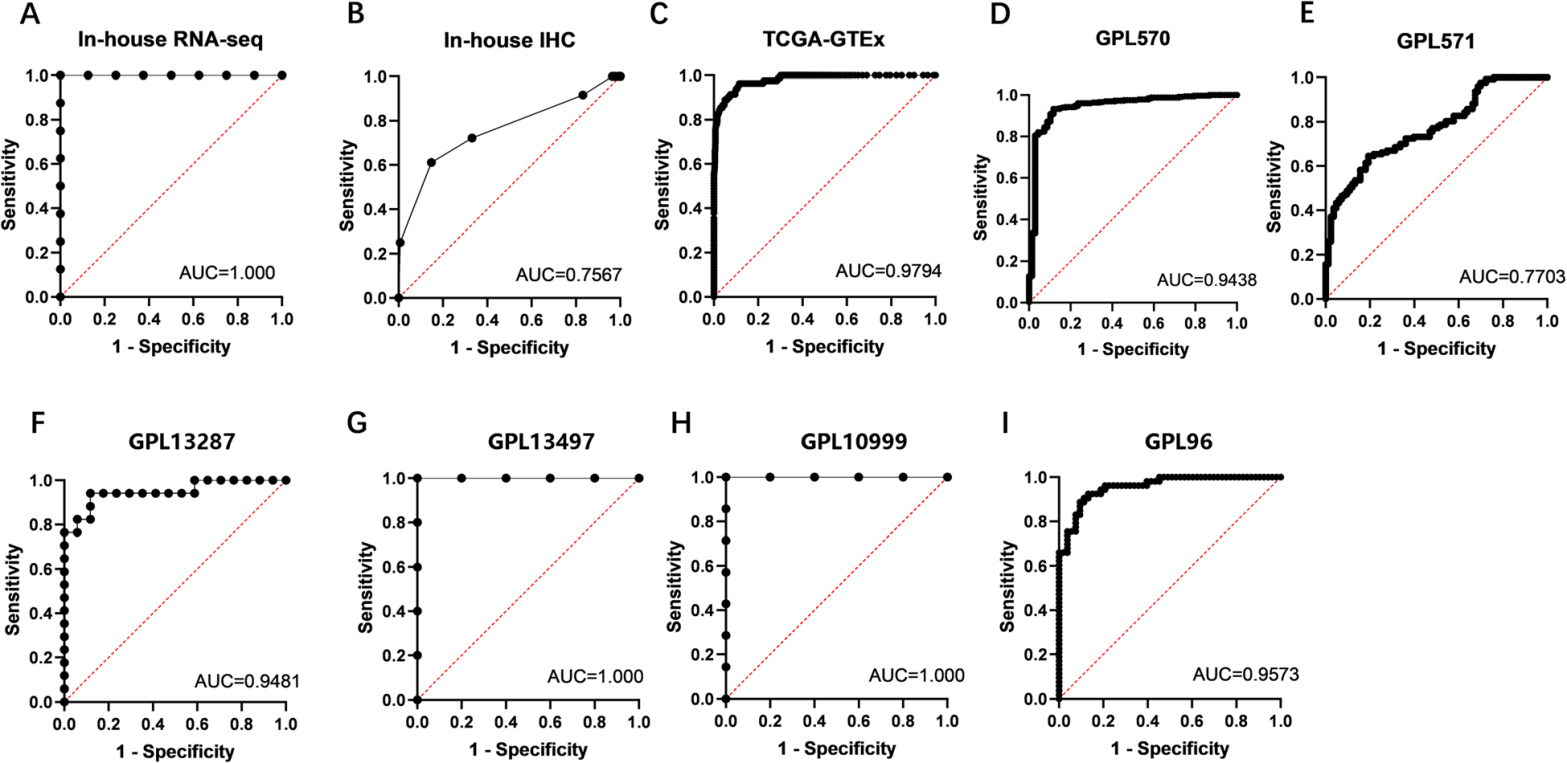
ROC curves of UBE2C mRNA and protein overexpression in ESCC tissues assessed by in-house RNA-seq, in-house IHC, TCGA-GTEx RNA-seq and tissue microarrays. a, ROC curve of UBE2C mRNA based on in-house RNA-seq; b, ROC curve of UBE2C protein based on in-house IHC; c, ROC curve of UBE2C mRNA based on TCGA-GTEx RNA-seq; d-I, ROC curve of UBE2C mRNA based on tissue microarrays

The average expression value of UBE2C mRNA was significantly higher in ESCC (5.8418 ± 0.56367) than in control (2.2041 ± 1.4291, *p* < 0.001) tissues (Fig. 2a). The AUC = 1 (Fig. 3a) indicated that UBE2C had a high diagnostic value in discriminating ESCC tissues from non-cancerous tissues.

### UBE2C protein was overexpressed in ESCC tissues

**Fig. 4:**
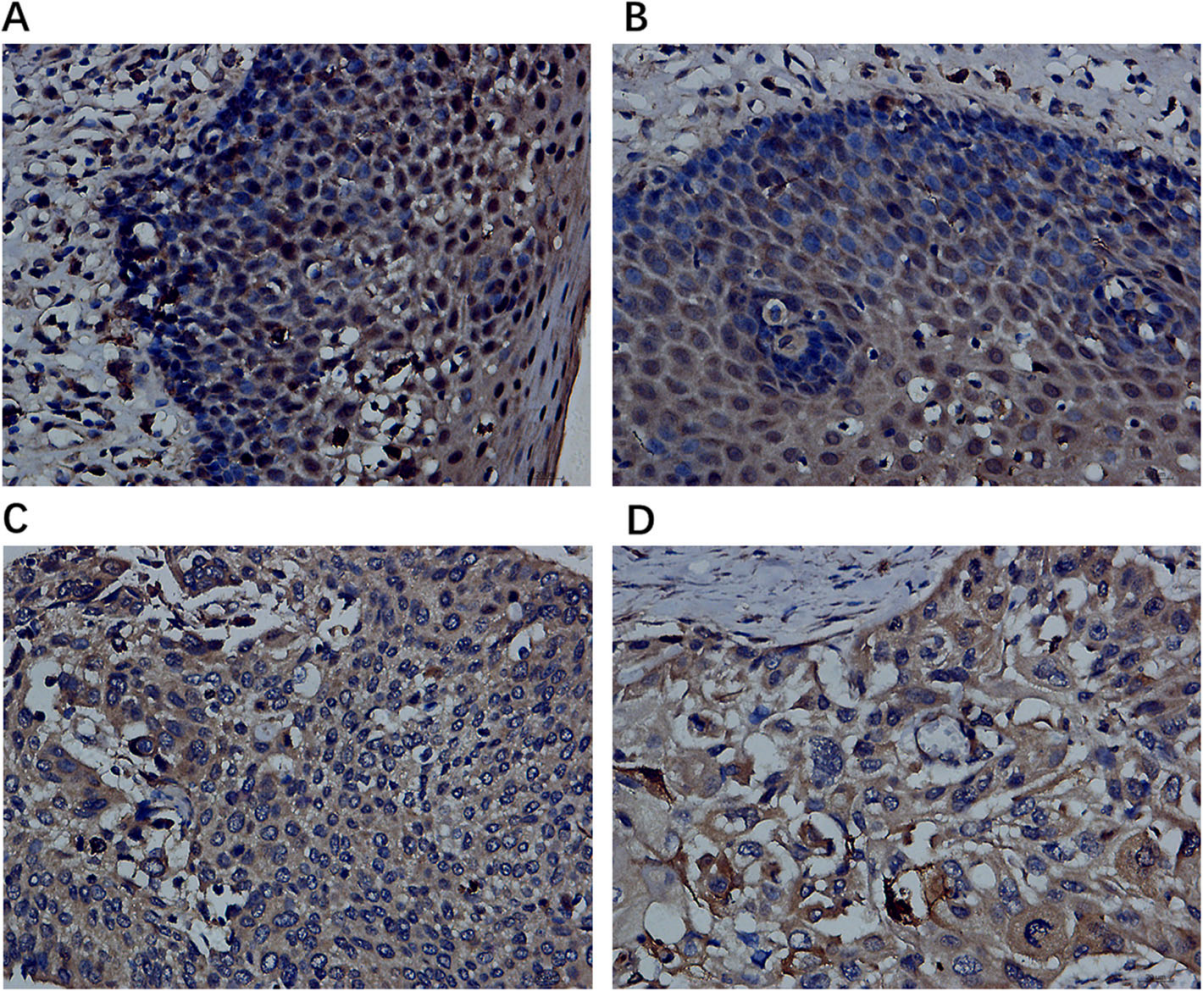
UBE2C protein overexpression in ESCC tissues assessed by in-house IHC. a-b, Normal esophageal tissue; c-d, ESCC tissue (400×)

The expression of UBE2C protein in ESCC and the relationship between UBE2C protein and UBE2C mRNA overexpression were analyzed by immunohistochemistry of 162 ESCC tissues and adjacent tissues. After removing the fragments, 144 cancer cases and 142 paracancer control cases were finally obtained. Among 144 cases of ESCC tissue, 104 (72%) showed positive expression of UBE2C protein (with a specified score of more than 6 being positive, and a score of less than or equal to 6 being negative), whereas only 47 (33%) of 142 adjacent tissues showed positive expression of UBE2C (*p* < 0.001) (Fig. 4a-d). Student’s t test revealed that the positive signal for UBE2C in ESCC was concentrated in the cytoplasm, with an average expression value (8.63889 ± 2.47112) significantly higher than in than non-cancer tissues (6.42958 ± 1.77611, *p <* 0.001) (Fig. 2b). The AUC = 0.7567 (Fig. 3b), indicating that UBE2C protein and mRNA were highly expressed in ESCC tissues.

### UBE2C mRNA was overexpressed in ESCC tissues in public data

The results of in-house RNA-seq and in-house IHC of ESCC tissues were verified by comparison of expression data from 82 ESCC cancer tissue samples and 1456 non-cancerous esophageal tissue controls from the ESCC dataset in the TCGA and the esophageal tissue in the GTEx. The average expression value of UBE2C was significantly higher in ESCC tissues (11.984 ± 0.874) than in the control tissues (6.077 ± 3.351, *p* < 0.001) (Fig. 2c). The AUC was 0.9794 (Fig. 3c), indicating that UBE2C could discriminate ESCC tissues from non-cancer tissues. We further expanded the sample size and improved the accuracy of the results, by including 15 tissue microarrays with UBE2C expression (GSE45670 (GPL570), GSE77861 (GPL570), GSE26886 (GPL570), GSE69925 (GPL570), GSE100942 (GPL570), GSE33810 (GPL570), GSE17351 (GPL570), GSE20347 (GPL571), GSE38129 (GPL571), GSE29001 (GPL571), GSE33426 (GPL571), GSE23400 (GPL96), GSE32424 (GPL10999), GSE45168 (GPL13497), GSE70409 (GPL13287)). For better analysis, we merging the microarrays from the same platform. GPL570 was merged by GSE45670 (GPL570), GSE77861 (GPL570), GSE26886 (GPL570), GSE69925 (GPL570), GSE100942 (GPL570), GSE33810 (GPL570), GSE17351 (GPL570), GSE45670 (GPL570), GSE77861 (GPL570), GSE26886 (GPL570), GSE69925 (GPL570), GSE100942 (GPL570), GSE33810 (GPL570) and GSE17351 (GPL570); GPL571 was merged by GSE20347 (GPL571), GSE38129 (GPL571), GSE29001 (GPL571) and GSE33426 (GPL571). The expression relationship of UBE2C in cancer tissues and control tissues was demonstrated by drawing violin diagrams and ROC curves. The average expression of UBE2C indicated significantly overexpression in ESCC tissues compared to control tissues in the 6 data sets after merging the same platform (Fig. 2d-i). The ROC curves of the six combined data sets revealed 5 of 6 datasets with AUC > 0.9 (Fig. 3d-i), suggesting significant differences in the expression of UBE2C in the ESCC versus the control group, and that the cancer tissues showed significantly high expression.

### Comprehensive analysis confirmed the UBE2C is overexpressed in ESCC

We integrated the RNA-seq and tissue microarray results from the public database (including 620 cases of ESCC and 1687 cases of non-cancerous tissue), and drew the combined SMD forest map and sROC curve. The value of SMD was 2.05 (95%CI: 1.57–2.52, *p* < 0.001), the AUC of the sROC curve was 0.91 (95%CI: 0.88–0.93), the sensitivity was 0.96 (95%CI: 0.81–0.99), and the specificity was 0.82 (95%CI: 0.73–0.89). We used Egger’s test and Begg’s funnel plot to visualize publication bias, but found no obvious publication bias for either the Begg’s (*p* = 0.548) or Egger’s (*p* = 0.325) tests. UBE2C was highly expressed in ESCC after comprehensive analysis) (data showed in supplementary information).

We further examined the mRNA expression level of UBE2C by combining the large throughput data from the public data with our in-house RNA-seq data (a total of 628 cancer tissues and 1695 healthy control tissues). The SMD of UBE2C mRNA expression values was 2.13 (95%CI: 1.66–2.60, *p* < 0.001), the AUC of sROC was 0.92 (95%CI: 0.90–0.94), the sensitivity was 0.96 (95%CI: 0.83–0.99), and the specificity was 0.84 (95%CI: 0.73–0.90). Begg’s test (*p* = 0.536), and Egger’s test (*p* = 0.201) showed no obvious publication bias. Therefore, in ESCC tissues, UBE2C mRNA was in a significantly upregulated state. (data showed in supplementary information).

**Fig. 5:**
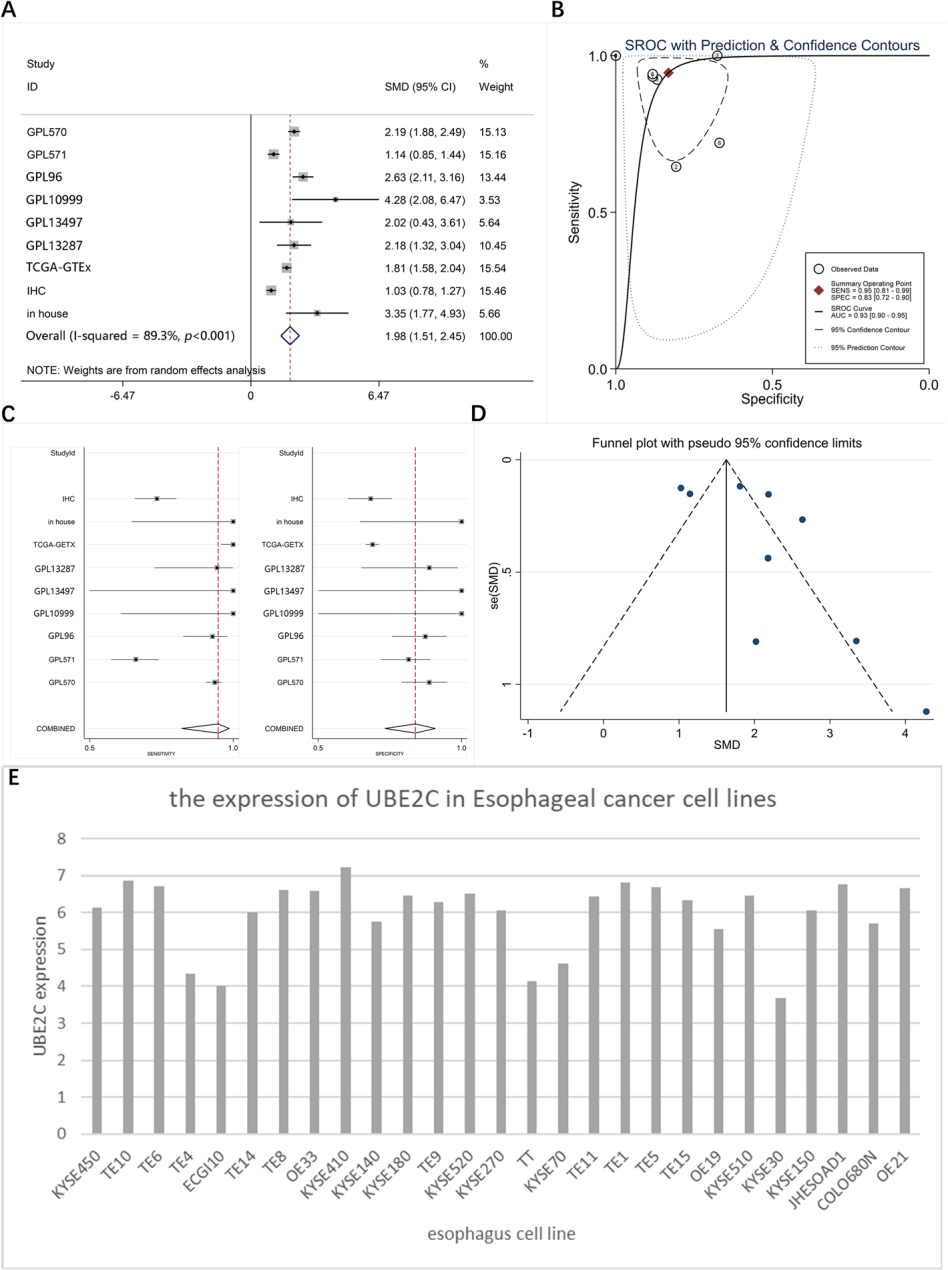
The comprehensive UBE2C expression level in ESCC and ESCC cell lines. a, Forest plot of UBE2C mRNA expression in ESCC based on in-house RNA-seq, in-house IHC, TCGA-GTEx RNA-seq and tissue microarrays. ESCC vs. normal, random-effects model; b, sROC of UBE2C in ESCC tissues based on in-house RNA-seq, in-house IHC, TCGA-GTEx RNA-seq and tissue microarrays; c, Sensitivity analysis of in-house RNA-seq, in-house IHC, TCGA-GTEx RNA-seq and tissue microarrays; d, Funnel plot of in-house RNA-seq, in-house IHC, TCGA-GTEx RNA-seq and tissue microarrays related to UBE2C; e, UBE2C mRNA expression of Esophageal cancer cell line based on CCLE database

We conducted a comprehensive evaluation of the UBE2C expression in ESCC by collecting the protein and mRNA expression data (including in-house RNA- seq, in-house IHC, TCGA-GTEx RNA-seq and tissue microarray), including 772 ESCC samples and 1837 control samples, to calculate a combined SMD and sROC. The heterogeneity among different research sources led us to choose the random effects model. The pooled SMD of UBE2C expression values was 1.98 (95% CI: 1.51–2.45, *p* < 0.001) (Fig. 5a), which indicated significant overexpression of UBE2C in ESCC tissues. Analysis of the diagnostic test results shows that the AUC of the sROC was 0.93 (95% CI: 0.90–0.95) (Fig. 5b), the sensitivity was 0.95 (95%CI: 0.81–0.99), and the specificity was 0.83 (95%CI: 0.72–0.90), indicating that UBE2C has a high accuracy in the diagnosis of ESCC versus non-carcinoma tissues. The sensitivity analysis shows no significant difference among all datasets (Fig. 5c). Funnel plot was used to calculate the publication bias (Fig. 5d). The Begg’s test (*p* = 0.602) and Egger’s test (*p* = 0.170) indicated no publication bias.

### UBE2C mRNA was expressed in esophageal cancer cell lines

The 27 esophageal cancer cell lines screened from CCLE (https://portals.broadinstitute.org/ccle) for further analysis of mRNA expression of UBE2C in ESCC all showed UBE2C expression (Fig. 5e).

### Correlation between UBE2C expression and the clinical characteristics in ESCC

Analysis of the clinical data of 82 cases of ESCC in the TCGA database revealed a significantly higher average UBE2C mRNA expression in the population younger than 60 or equal to 60 years old (12.2077 ± 0.78864) than that in persons elder than 60 years old (11.6763 ± 0.91481, *p* = 0.007). No significant correlation was noted between UBE2C expression and other clinicopathological parameters.

**Fig. 6:**
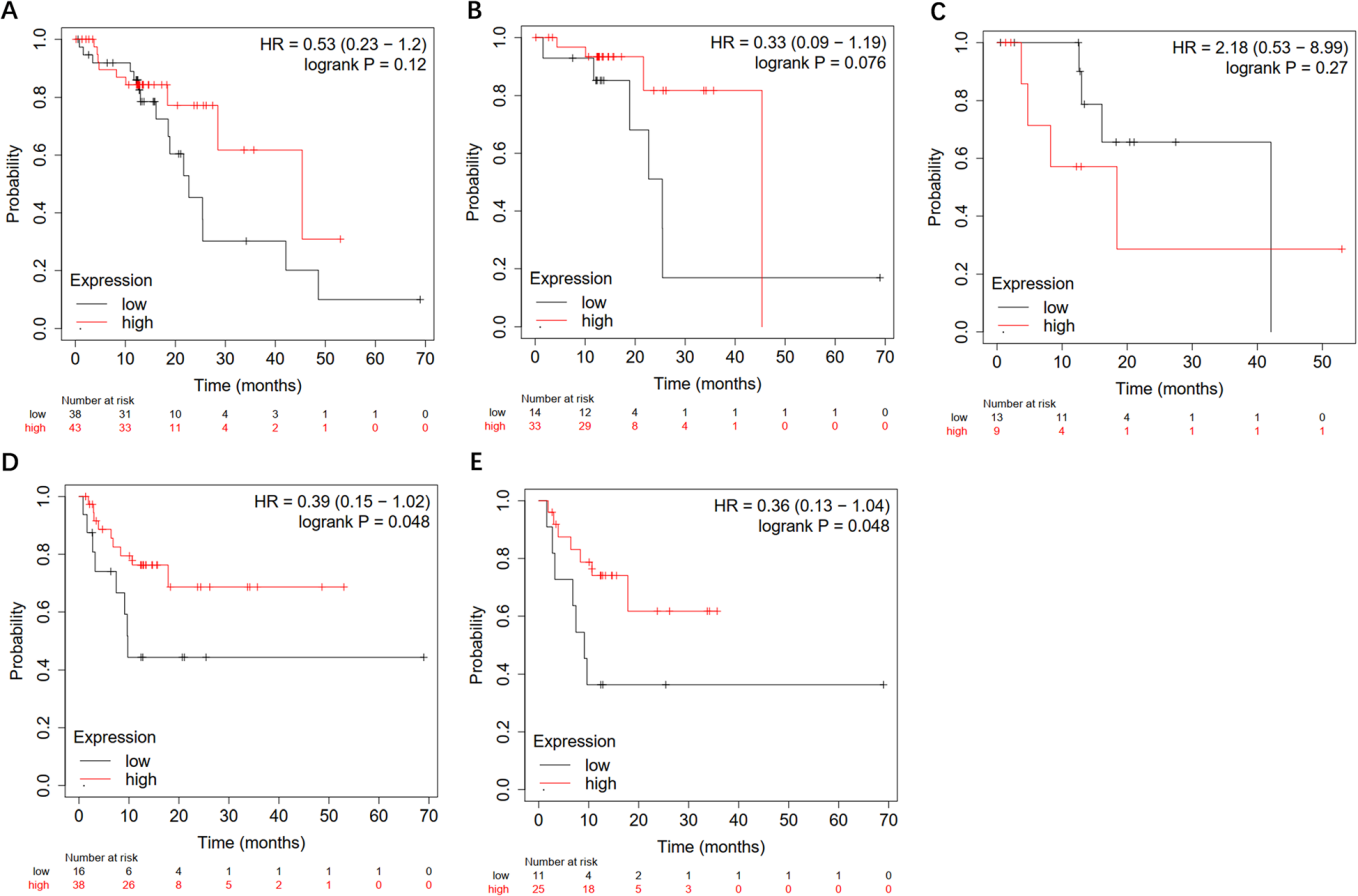
Kaplan-Meier survival analysis of UBE2C in ESCC based on Kaplan Meier-plotter databases. a, OS survival analysis of UBE2C in ESCC in all stage; b, OS survival analysis of UBE2C in ESCC in stage II; c, OS survival analysis of UBE2C in ESCC in stage III; d, RFS survival analysis of UBE2C in ESCC in all stage; e, RFS survival analysis of UBE2C in ESCC in stage II

Results of survival analysis showed that ESCC patients with high expression of UBE2C tended to have higher overall survival (OS), and *p* value was not statistically significant (Fig. 6a). Interestingly, through subgroup analysis of ESCC patients at different stages, we found that in early stage of ESCC patients (stage II), patients with high expression of UBE2C tended to have higher OS, and *p* value was not statistically significant (Fig. 6b). However, in advanced stage of ESCC (stage III), patients with high expression of UBE2C had a lower five-year survival rate, and *p* value was not statistically significant (Fig. 6c). This may indicate that UBE2C plays a different role in different stages of ESCC. In addition, by analyzing the relapse-free survival (RFS) of UBE2C in the ESCC dataset, we found that ESCC patients with high expression of UBE2C tended to have a lower risk of recurrence, and the *p* value was not statistically significant (Fig. 6d-e).

### Analysis of the UBE2C copy number

The copy number analysis of UBE2C in the esophageal cancer dataset showed that UBE2C was amplified in 32 (6%) of 559 cases (Fig. 8d).

### Functional analysis for the DEGs of ESCC and UBE2C related-genes

**Fig. 7:**
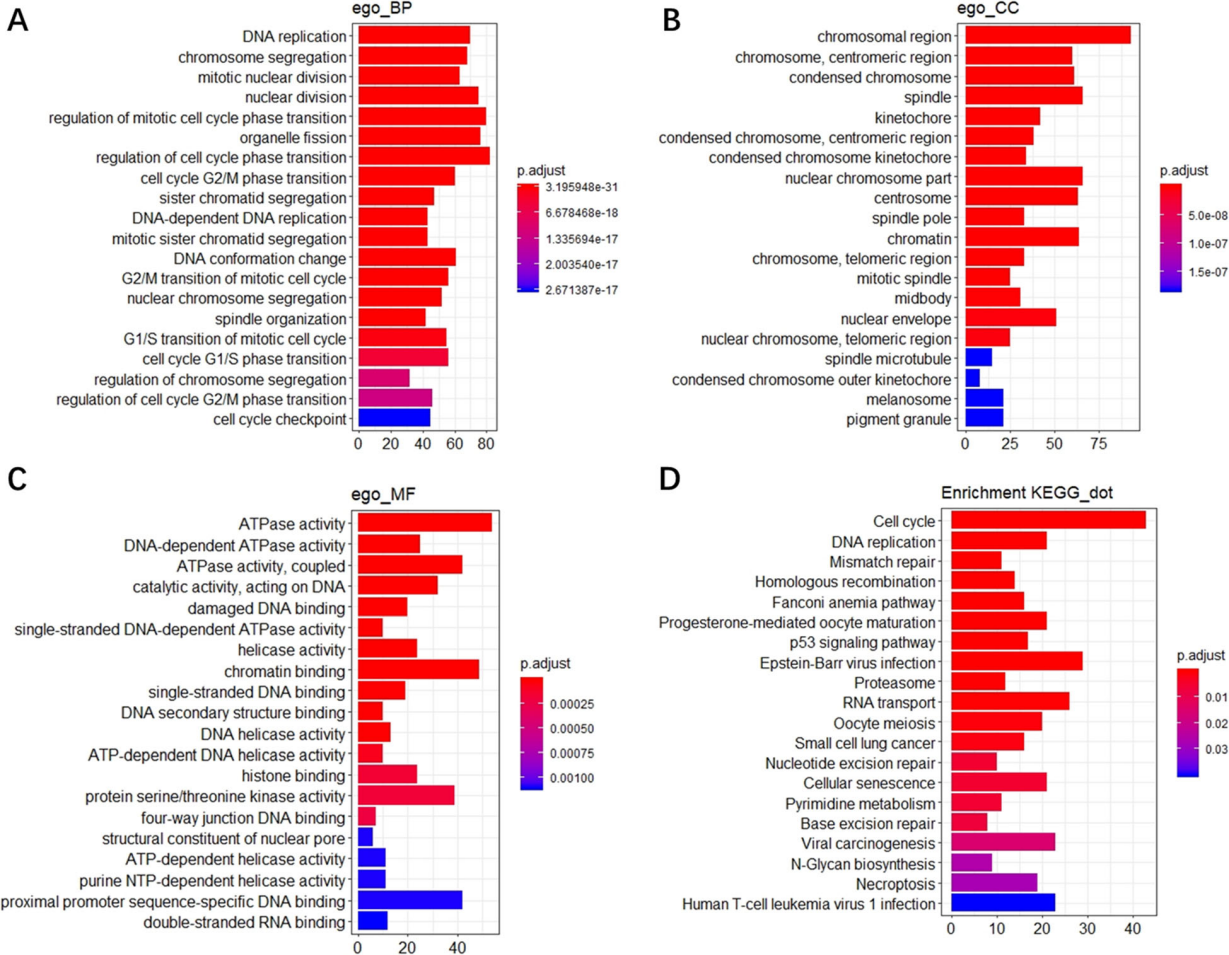
GO and KEGG analyses of 890 overlapped DEGs and related-genes. a, Biological process (BP); b, Cellular component (CC); c, Molecular function (MF); d, KEGG pathway annotations

**Fig. 8:**
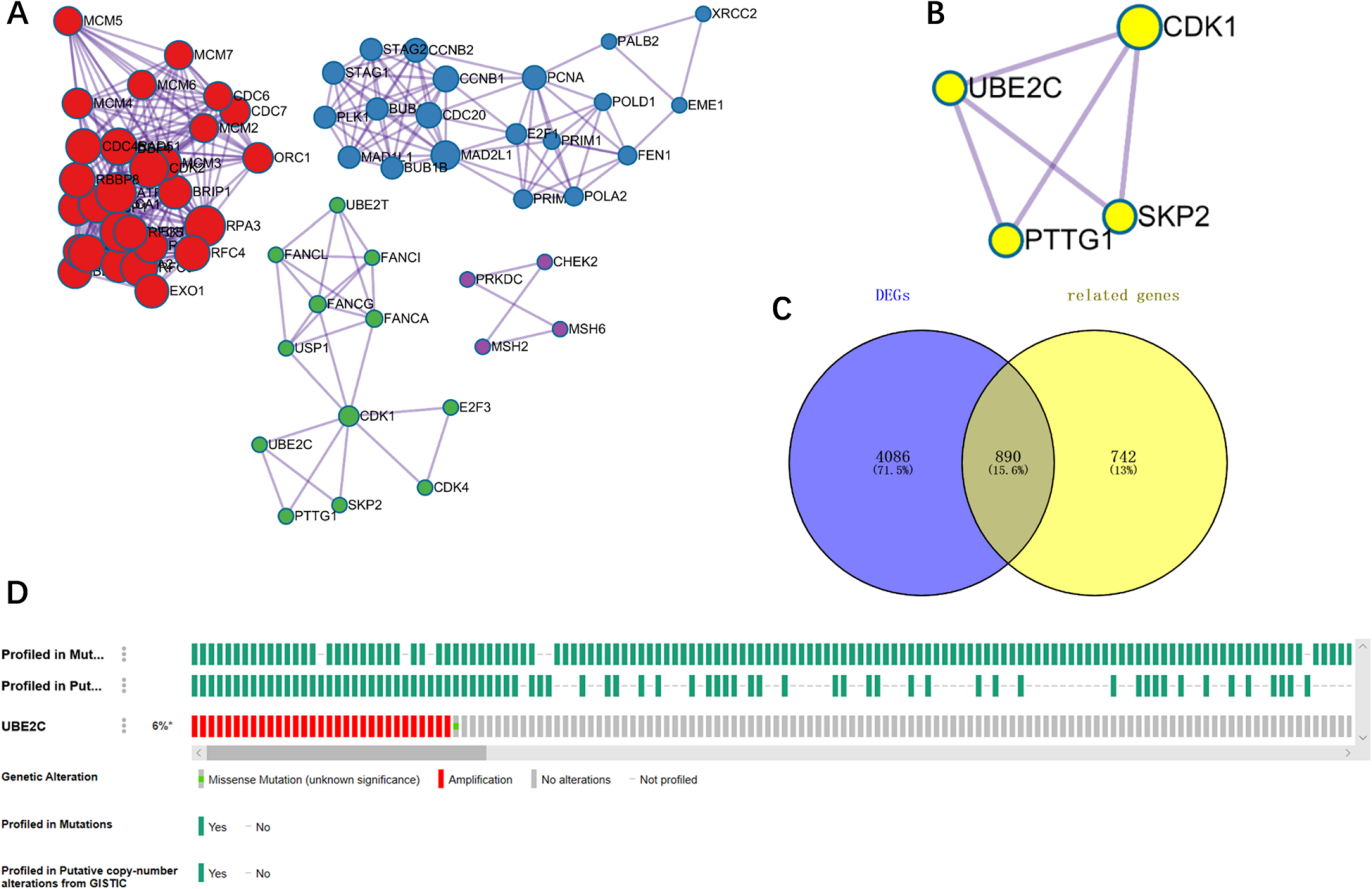
a, PPI network of the top five KEGG pathways; b, the related protein of UBE2C in PPI network; c, Venn diagram of the overlapped DEGs and UBE2C related genes; d, Copy number variation of UBE2C based on esophageal cancer

The mechanism of UBE2C in ESCC was further analyzed by selecting 4976 genes significantly differentially expressed in ESCC and 1632 genes significantly related with UBE2C by RRA combined with artificial ranking. Ultimately, 890 genes (the intersection of DEGs and UBE2C related genes) (Fig. 8c) were selected for GO and KEGG analysis. The GO analysis showed that the biological processes of DEGs and UBE2C related genes were focused primarily on DNA replication, chromosome segregation, and mitotic nuclear division (Fig. 7a). The main cellular components included the chromosomal region, chromosome, centromeric region, condensed chromosome spindle, and kinetochore (Fig. 7b). The molecular functions included ATPase activity, DNA-dependent ATPase activity, ATPase activity, coupled catalytic activity acting on DNA, and damaged DNA binding (Fig. 7c). The KEGG pathway analysis indicated that these genes were mainly involved in pathways such as the cell cycle and DNA replication (Fig. 7d). The PPI results of the first five KEGG pathway proteins (Table 1) showed that UBE2C mainly influenced the biological function of esophageal cancer by synergistic effects with CDK1, PTTG1, and SKP2 (all three are involved in the cell cycle pathway) (Fig. 8a-b).

**Table 1:** Genes for the top five KEGG pathways

KEGG pathways	Gene symbol
Cell cycle	BUB1B, CCNA2, CCNB1, CCNB2, CDC20, CDC25B, CDC25C, CDC6, E2F3, MAD2L1, MCM2, MCM3, PLK1, PRKDC, TTK, BUB1, CCNE2, CDC45, CDC7, CDK1, CHEK1, CHEK2, DBF4, E2F1, MAD1L1, MAD2L2, MCM4, MCM5, MCM6, MCM7, PCNA, PKMYT1, RBL1, SKP2, GSK3B, ATR, STAG2, PTTG1, STAG1, CDC25A, CDK2, CDK4, ORC1
DNA replication	RFC4, DNA2, MCM2, MCM3, RNASEH2A, FEN1, LIG1, MCM4, MCM5, MCM6, MCM7, PCNA, POLD1, PRIM1, PRIM2, RFC3, RPA3, RFC5, RFC2, POLA2, POLE3
Mismatch repair	RFC4, EXO1, MSH6, LIG1, PCNA, POLD1, RFC3, RPA3, RFC5, RFC2, MSH2
Homologous recombination	BLM, BRCA1, RAD51, XRCC2, EME1, PALB2, POLD1, RAD54B, RAD54L, RBBP8, RPA3, TOPBP1, BRCA2, BRIP1
Fanconi anemia pathway	FANCA, UBE2T, BLM, BRCA1, FANCI, RAD51, EME1, FANCG, PALB2, RPA3, BRCA2, RMI1, ATR, BRIP1, FANCL, USP1

### Prediction of miRNA and lncRNA

**Fig. 9:**
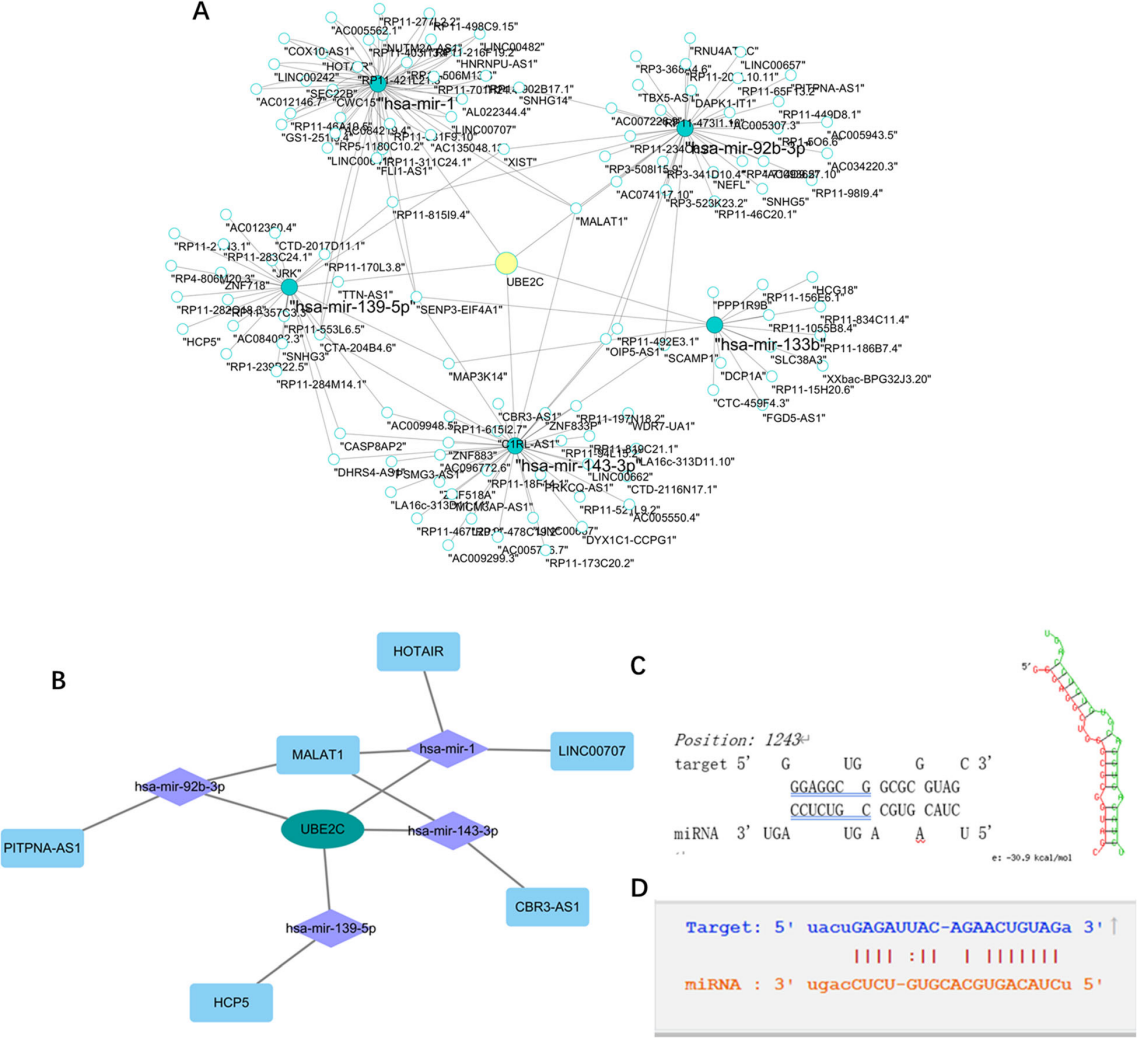
a, putative ceRNA network; b, putative ceRNA network; c, The binding site between hsa-miR-139-5p and UBE2C; d, The binding site between hsa-miR-139-5p and HCP5

The potential miRNAs upstream of UBE2C were explored using a variety of miRNA online databases (miRWalk, miRanda, miRDB, miRNAMap, PITA, RNAhybrid, Microt4, mirbridge, miRMap, Pictar2, RNA22, Targetscan). The predicted miRNAs were intersected with the differential miRNAs in at least two miRNA datasets among TCGA, GPL16543 (GSE43732), GPL18402 (GSE71043), GPL23365 (GSE112840), GPL24967 (GSE114110), and GPL16770 (GSE59973). The 14 miRNAs obtained in the intersection were used to predict the upstream lncRNAs through the miRNet database (Fig. 9a) and intersected with the differentially expressed lncRNAs in the ESCC datasets (Fig. 9b) (including the combined TCGA-GTEx, GPL570, GPL571, GPLl96 (GSE2340), GPL10999 (GSE32424), GPL13497 (GSE45168), GPL13287 (GSE70409), and GPL13607 (GSE45350). Finally, six candidate lncRNAs were obtained (Table 2).

**Table 2:** putative miRNA and lncRNA

putative miRNA	putative miRNA	putative lncRNA
hsa-miR-4419b	hsa-miR-671-5p	HCP5
hsa-miR-378g	hsa-miR-29b-2-5p	MALAT1
hsa-miR-3154	hsa-miR-139-5p	HOTAIR
hsa-miR-663b	hsa-miR-1	CBR3-AS1
hsa-miR-143-3p	hsa-miR-143-5p	LINC00707
hsa-miR-486-3p	hsa-miR-133b	PITPNA-AS1
hsa-miR-92b-3p	hsa-let-7d-3p	

### Construction of the ceRNA network

The possible targeting relationship among lncRNA, miRNA, and UBE2C in ESCC tumor tissue was further clarified by calculating the combined SMD of the candidate miRNA and lncRNA expression values. We narrowed the scope by choosing miRNAs that were negatively correlated with UBE2C expression as an example, and we found that only HCP5/has-miR-139-5p/UBE2C expression showed a consistent relationship. In addition, the binding sites of hsa-miR-139-5p and UBE2C were obtained from RNAhybrid database (Fig. 9c), and of hsa-miR-139-5p and HCP5 were from Starbase database (Fig. 9d).

**Fig. 10:**
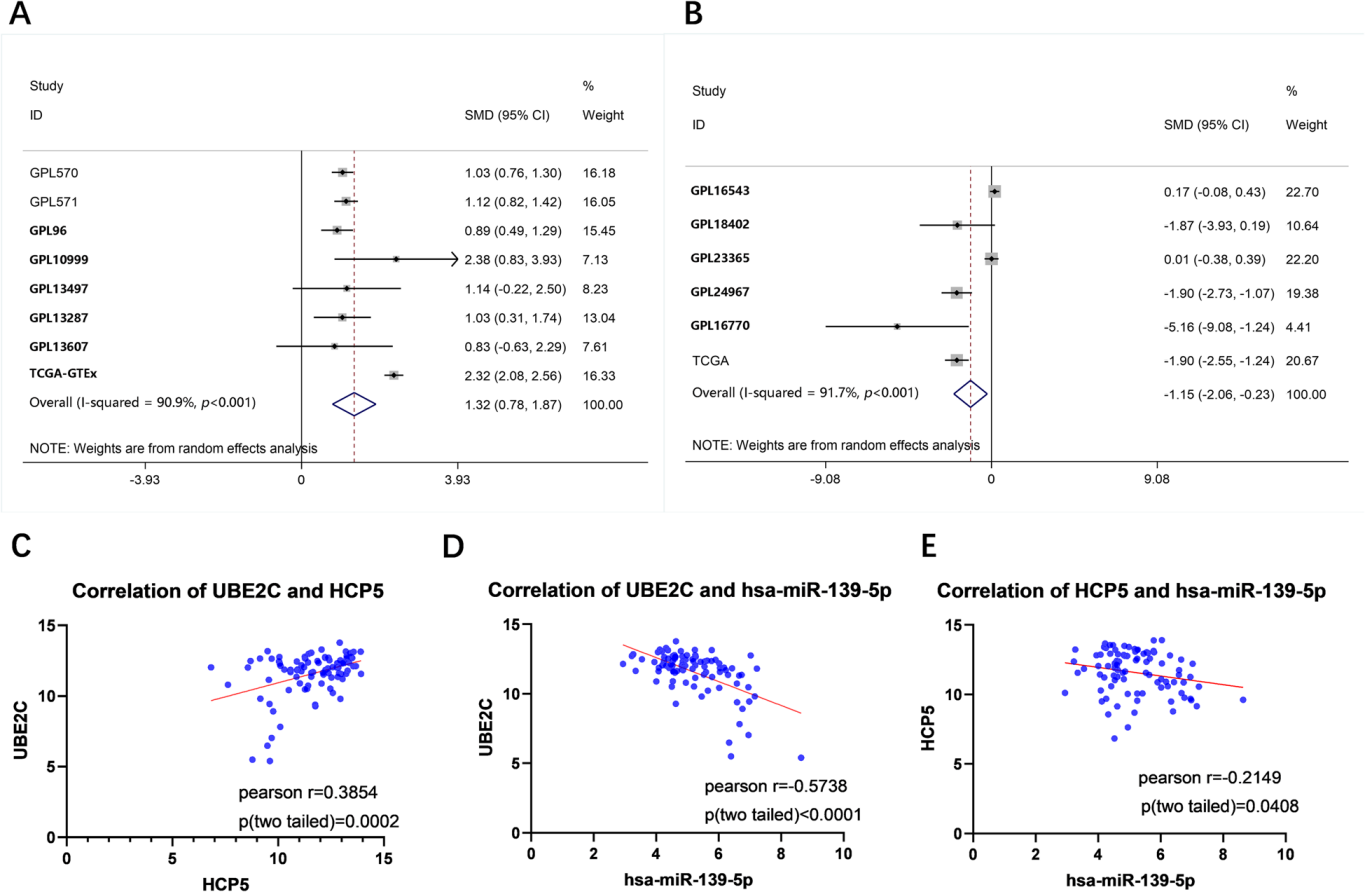
Forest plot of HCP5 and hsa-miR-139-5p expression and correlation among HCP5, hsa-miR-139-5p and UBE2C in ESCC based on included microarray. a, Forest plot of HCP5 expression in ESCC based on included microarray; b, Forest plot of hsa-miR-139-5p expression in ESCC based on included microarray; c, correlation between UBE2C and HCP5; d, correlation between UBE2C and hsa-miR-139-5p; e, correlation between HCP5 and hsa-miR-139-5p

The pooled SMD of lncRNA HCP5 in ESCC was 1.32 (95%CI: 0.78–1.87, *p* < 0.001, using the random effects model) (Fig. 10a), indicating that lncRNA HCP5 was highly expressed in ESCC. The pooled SMD of miRNA hsa-miR-139-5p in ESCC tissue was − 1.61 (95%CI: − 3.20 to − 0.02, *p <* 0.001, using the random effects model). The SMD of miRNA hsa-miR-139-5p expression in human tissues (including cancer tissues and body fluids of ESCC patients) was - 1.15 (95% CI: − 2.06–0.23, *p <* 0.001, using the random effects model) (Fig. 10b), indicating that miRNA hsa-miR-139-5p was downregulated in ESCC tissues.

**Fig. 11:**
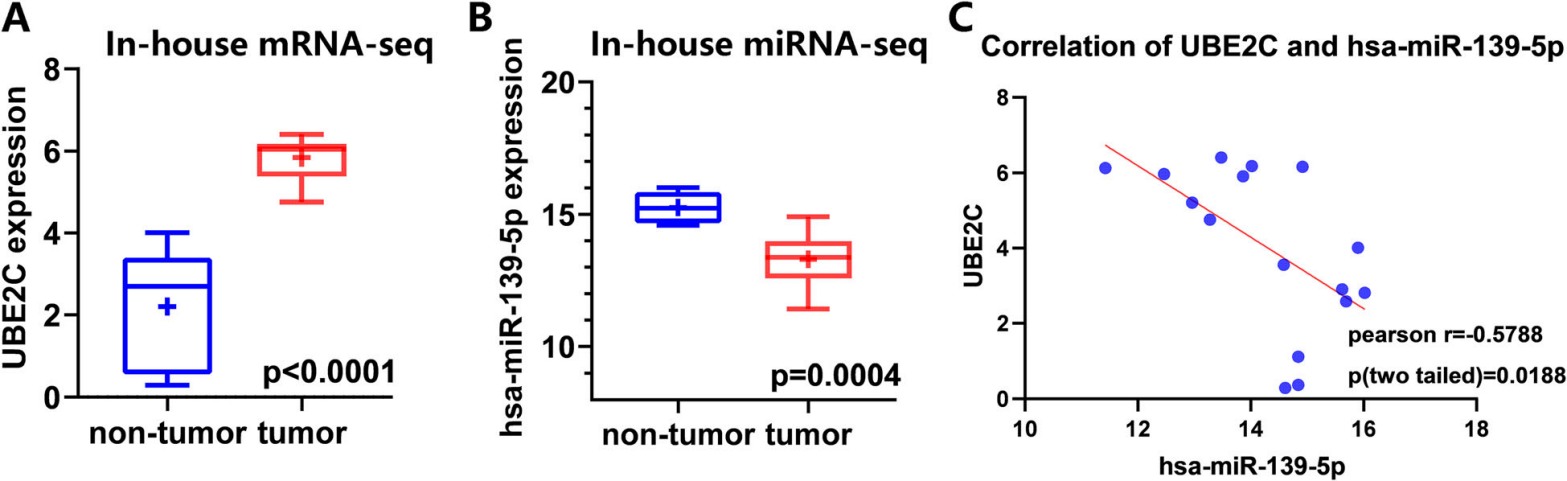
a, Box plot of UBE2C mRNA expression based on in-house RNA-seq; b, Box plot of hsa-miR-139-5p miRNA expression based on in-house RNA-seq; c, correlation between UBE2C and hsa-miR-139-5p based on in-house RNA-seq

The results of correlation analysis based on TCGA suggested a significant positive correlation between lncRNA HCP5 and mRNA UBE2C (Pearson *r =* 0.3854, *p* = 0.0002) (Fig. 10c) and a significant negative correlation between miRNA hsa-miR-139-5p and mRNA UBE2C (Pearson *r =* − 0.5738, *p* < 0.0001) (Fig. 10d), as well as lncRNA HCP5 (Pearson *r =* 0.2149, *p* = 0.0408) (Fig. 10e). Moreover, the UBE2C and hsa-miR-139-5p analysis based on in-house RNA-seq showed that the average expression value of UBE2C mRNA was significantly higher in ESCC (5.8418 ± 0.56367) than in control (2.2041 ± 1.4291, *p* < 0.0001) tissues. And the average expression value of hsa-miR-139-5p miRNA was significantly lower in ESCC (13.3007 ± 1.0573) than in control (15.2635 ± 0.6011, *p <* 0.001) tissues. The correlation analysis suggested a significant negative correlation between UBE2C and hsa-miR-139-5p (Pearson *r =* − 0.5788, *p* = 0.0188). (Fig. 11a-c) This was basically consistent with the correlation results of UBE2C and hsa-miR-139-5p in TCGA (Pearson *r =* − 0.5738, *p <* 0.0001).

## Discussion

Our analysis of the expression of UBE2C in 772 ESCC tissue samples and 1837 non-cancerous tissue control samples, which is the largest sample size analyzed to date, confirmed a significantly high expression of UBE2C in ESCC tissue. We also verified lncRNA HCP5 expression in 624 ESCC tissues and 1691 non cancer control tissues. In the same way, miRNA hsa-miR-139-5p was verified in 119 ESCC human tissues and 199 healthy controls. Together with our GO and KEGG analysis of UBE2C related genes and ESCC differential genes, we were able to construct a ceRNA network related to UBE2C in ESCC. Based on our findings, we speculate that this ceRNA axis consisting of HCP5/hsa-miR-139-5p/UBE2C may exist in ESCC. That is, lncRNA HCP5, as a competitive endogenous RNA, adsorbs the hsa-miR-139-5p miRNA, thereby regulating the downstream UBE2C and affecting the biological function of ESCC. Of course, this hypothesis needs further experimental confirmation.

Esophageal cancer is particularly prevalent in China, and ESCC is the most common pathological type. The early symptoms of ESCC are insidious and difficult to detect, and most patients seek medical help due to dysphagia. Unfortunately, this only occurs during the middle and advanced stages of ESCC, so the first diagnosis occurs at a late stage [13]. The treatment of esophageal cancer is mainly based on a combination of traditional surgery and radiotherapy/chemotherapy. Some new progress has been made in multidisciplinary comprehensive treatment and imaging diagnosis in recent years, but the 5-year survival rate for esophageal cancer is much lower than 30% due to its invasiveness and pronounced metastasis ability, as well as the difficulty in early diagnosis [2, 14, 15]. UBE2C is generally considered as an important tumor biomarker, as it is overexpressed in breast cancer, thyroid cancer, lung cancer, gastric cancer, and other tumor tissues, but shows low expression in normal tissues. The expression level of UBE2C has also been related to the aggressiveness of the tumor [3]. A higher UBE2C expression generally predicts a lower survival rate and a higher risk of recurrence. High UBE2C expression has also been was associated a highly malignant tumor phenotype [16–19]. However, research on UBE2C in ESCC has not been sufficiently comprehensive.

The integration of large numbers of sample data in the present study confirmed the high expression of UBE2C in ESCC, as well as a significant correlation between expression and gender (The expression level of UBE2C was significantly higher in males than in females). Interestingly, several studies have verified the expression level of UBE2C in ESCC through various detection methods. For instance, Palumbo et al. demonstrated an upregulated expression of UBE2C mRNA and protein in ESCC tissues through qRT-PCR and immunohistochemical analysis of ESCC and control tissue specimens and further confirmed, with in vitro experiments, that UBE2C affected the proliferation of ESCC cells by interfering with the level of cyclin B1 [4]. Similarly, Nicolau-Neto et al. proposed that the expression of UBE2C in ESCC may be regulated by transcription factor FOXM1 and that upregulation of UBE2C may affect the occurrence of tumors by acting on cell cycle pathways, a common phenomenon in human tumors [20]. Another in vitro study confirmed that UBE2C knockdown significantly inhibited proliferation and induced apoptosis of the TE1 esophageal cancer cell line [21]. These previous studies further confirm the reliability of our results. In addition, through the survival analysis of UBE2C in the ESCC dataset, we found that UBE2C is likely to play different roles in different stages of the ESCC. Moreover, a 6% copy number amplification of UBE2C gene was observed, although no correlation between amplification and prognosis was found. We speculate that UBE2C can be used as a diagnostic and prognostic indicator to assist in the early diagnosis and assessment of ESCC patients.

Several studies have confirmed the significantly high expression of UBE2C in ESCC, but the role of UBE2C in ESCC has not yet been determined. In the present study, RRA combined with artificial ranking was used to select the intersections of ESCC differential genes and UBE2C related genes for GO and KEGG analysis. Construction of a PPI network graph indicated that UBE2C may synergistically regulate the cell cycle pathway through PTTG1, CDK1 and SKP2 (proteins of the cell cycle pathway) and that this may be the mechanism by which UBE2C performs its multifunctional and key biological functions in ESCC. Tumors are characterized by abnormal growth and uncontrolled proliferation of cells and the abnormalities of cell proliferation, differentiation, and apoptosis are all involved in the occurrence and development of tumors. The cell cycle is a continuous and accurate process, so cell cycle disorder is the most important mechanism of tumor formation. The current viewpoint is that UBE2C mainly influences the occurrence and development of tumors by participating in cell cycle pathways [4, 20–23]. Consequently, UBE2C may also participate in the occurrence and development of ESCC through regulation of cell cycle pathways.

In recent years, increasing evidence has pointed to lncRNAs, miRNAs, and other non-coding RNAs as key regulators of various biological processes and have postulated a post-transcriptional regulatory role in controlling cell differentiation, gene expression, cell cycle, apoptosis, and other functions driving the pathogenesis of tumors [24]. The competing endogenous RNA (ceRNA) hypothesis has since revealed a brand new regulatory mechanism for gene expression, with lncRNA, miRNA, and mRNA as important components that interact with each other through microRNA response elements (MREs). The target mRNAs of miRNAs are a class of RNA that can encode proteins. The 3′-UTR of mRNA mostly contains highly conserved regions bound to miRNA seed sequences and can therefore be regulated by miRNAs. Although lncRNA cannot encode proteins, it can be used in ceRNA networks to compete with mRNA in binding the same miRNA, thereby regulating the target gene [5].

The ceRNA hypothesis has attracted the attention of many scholars and researchers since it was first proposed. Currently, ceRNA is regarded as a biomarker of cancer, as a growing body of evidence suggests that ceRNA, consisting of lncRNA/miRNA/mRNA networks, plays key roles in a variety of human cancers, including breast, stomach, liver, and pancreatic tumors [25]. The findings of the present study now indicate an involvement of ceRNA in ESCC as well.

A new triple regulation network of lncRNA/miRNA/mRNA was successfully constructed in the present study by combining multiple miRNA and lncRNA online prediction databases with tissue microarrays and RNA-seq data. In this regulatory network, miRNA is significantly down-expressed in both ESCC cancer tissues and human tissues (including humoral tissues) from ESCC patients. In addition, lncRNA HCP5 was significantly positively correlated with UBE2C mRNA in this network, and both were significantly negatively correlated with miRNA. Interestingly, HCP5 has been reported as a ceRNA with involvement in the development of lymphoma [26], oral squamous cell carcinoma [27], breast cancer [28], laryngeal squamous cell carcinoma [29], and prostate cancer [30].

Other studies have also shown that the expression of has-miR-139-5p is downregulated in ESCC tissues, suggesting that it may be a promising biomarker for early screening of high-risk groups and for early detection of ESCC [31]. Interestingly, Shuo has also confirmed in his doctoral thesis that the expression of has-miR-139-5p was downregulated in ESCC tissues. Overexpression of mir-139-5p was shown to inhibit the proliferation of ESCC cells, to lead to cell cycle arrest, to inhibit the migration and invasion of tumor cells, and to inhibit tumor formation in nude mice. This evidence further enhances the reliability of the results of this study. However, one point to consider regarding the present study is that the ceRNA hypothesis indicates that the expression level of miRNA may be upregulated, downregulated, or unchanged. In order to narrow the scope, miRNA with significantly downregulated expression level in ESCC was selected in the present study to construct the ceRNA network, but this does not mean that other regulatory axes do not also exist. We speculate that a HCP5/has-miR-139-5p/UBE2C axis may also exist in ESCC. LncRNA HCP5 can bind to has-miR-139-5p through miRNA regulatory elements and play a sponge-like role in absorbing miRNAs, thereby releasing the inhibition of miRNAs on the target UBE2C gene and affecting the occurrence and development of ESCC. However, the results need to be confirmed by further in vitro experiments.

In addition, our study has some limitations. One is that the researchers who conducted the included studies did not agree on the location of UBE2C expression or on the evaluation, test methods, and defined values of positive and negative, which may affect the accuracy of our results. A second limitation is that although our results were obtained by integrating and analyzing a large number of existing data and combining the in-house immunohistochemistry and RNA-seq data of ESCC patients with a high degree of credibility, we still need further in vivo or in vitro experiments to study its function. Another limitation was that fluid detection has the potential to help the early diagnosis of tumors; therefore, expression of UBE2C in the body fluids of ESCC patients and non-ESCC healthy people needs to be further studied. A fourth limitation is that although the ceRNA axis constructed in this study is based on the expression of lncRNA, miRNA and mRNA in ESCC, as well as in silico analysis, which has high reliability, further in vitro and in vivo experimental verification is still needed to provide a more reliable basis for future research and clinical practice.

## Conclusion

In summary, this study indicates that UBE2C is overexpressed in ESCC tissues and that the high expression of UBE2C may influence the biological function of ESCC by regulating the cell cycle. We also constructed a UBE2C-related ceRNA network for ESCC (HCP5/hsa-miR-139-5p/UBE2C). Our study may provide new strategies for the clinical diagnosis and treatment of esophageal cancer.

## Supplementary Information

**Additional file 1.**

**Additional file 2.**

## Data Availability

The in-house RNA-seq data is deposited in the Gene Expression Omnibus (GEO) (https://www.ncbi.nim.nih.gov/geo/) repository, accession number (GSE164158). The rest data comes from TCGA, GTEx, GEO, CCLE, cBioPortal and KM-plotter databases, which are all public open platforms.

## Abbreviations

ESCC: Esophageal squamous cell carcinoma
UBE2C: Ubiquitin Conjugating Enzyme E2 C
ceRNA: Competitive endogenous RNA
TPM: Transcripts Per Million
IHC: Immunohistochemistry
TCGA: The Cancer Genome Atlas
GTEx: Genotype-Tissue Expression
GEO: Gene Expression Omnibus
CCLE: The Cancer Cell Line Encyclopedia
ROC: Receiver operating characteristic curve
AUC: Area under the curve
SMD: Standardized mean difference
CNV: Copy number variation
DEGs: Differentially expressed genes
RRA: Robust rank aggregation
GO: Gene ontology
KEGG: Kyoto Encyclopedia of Genes and Genomes
PPI: Protein-Protein Interaction
OS: Overall survival
RFS: Relapse-free survival
