# Supplementary material for: Overexpression of UBE2C in esophageal squamous cell carcinoma tissues and molecular analysis
Source: BMC Cancer. 2021 Sep 6;21:996. doi: 10.1186/s12885-021-08634-6 (PMC8422647; doi:10.1186/s12885-021-08634-6)
Supplement: Supplementary file 1 — Additional file 1. [file 12885_2021_8634_MOESM1_ESM.pdf]

Table S1 Expression of UBE2C in ESCC.

Accession	Exp n	Exp mean	Exp sd	Ctrl n	Ctrl mean	Ctrl sd	p value	AUC
GPL570	329	9.669028	0.777051	68	7.972496	0.772762	<0.001	0.9438
GPL571	127	9.025992	1.319753	83	7.534212	1.276483	<0.001	0.7703
GSE23400GPL96	53	9.549963	0.54492	53	8.2421	0.442618	<0.001	0.9573
GSE32424	7	4.402059	0.588172	5	1.548651	0.771019	<0.001	1
GSE45168	5	12.44534	0.316927	5	9.7075	1.891234	0.012755	1
GSE70409	17	11.30276	1.234927	17	8.9822	0.861644	<0.001	0.9481
TCGA-GETX	82	11.98372	0.874241	1456	6.076742	3.350544	<0.001	0.9794
in house RNA-seq	8	5.841758	0.563671	8	2.204144	1.4291	<0.001	1
in house IHC	144	8.638889	2.471123	142	6.429577	1.776114	<0.001	0.7567

Table S2 Expression of HCP5 in ESCC.

Accession	Exp n	Exp mean	Exp sd	Ctrl n	Ctrl mean	Ctrl sd	p value
GPL570	329	8.555206	1.048541	68	7.479011	1.017334	<0.001
GPL571	127	8.082094	1.150129	83	6.7176	1.31579	<0.001
GSE23400GPL96	53	8.05	0.75465	53	7.497087	0.44321	<0.001
GSE32424	7	11.62143	4.73652	5	2.806	0.819225	0.002
GSE45168	5	8.7137	0.601044	5	7.9568	0.724019	0.11
GSE70409	17	12.6597	1.627955	17	11.3129	0.89266	0.005317
GSE45350	4	10.11665	1.043032	4	9.4875	0.24832	0.285
TCGA-GETX	82	11.7706	1.45737	1456	9.469327	0.958751	<0.001

Table S3 Expression of hsa-miR-139-5p in ESCC.

Accession	Exp n	Exp mean	Exp sd	Ctrl n	Ctrl mean	Ctrl sd	p value
GSE43732	119	-4.5982	3.777475	119	-5.0692	0.481332	0.178585
GSE71043	3	-3.31519	0.009805	3	-3.1974	0.08861	0.084083
GSE112840	52	1.5035	2.01996	52	1.492885	1.97522	0.979
GSE114110	30	-5.3308	2.82095	10	-0.6218	0.511748	<0.001
GSE59973	3	-7.64201	0.520969	3	-5.24623	0.399646	0.003
TCGA	95	5.0254	0.903825	12	6.7158	0.783637	<0.001

Table S4 Information of included RNA-seq and microarray.

Author	Year	Country	Data source	Test method/Platform	Sample type	RNA type
		China	IHC	NR	Tissue	mRNA/lncRNA
		China	RNA-seq	NR	Tissue	mRNA/lncRNA
TCGA		USA	TCGA	NR	Tissue	mRNA/lncRNA
Jing Wen et al	2014	China	GSE45670	Affymetrix GPL570	Tissue	mRNA/lncRNA
Kory R Johnson et al	2017	USA	GSE77861	Affymetrix GPL570	Tissue	mRNA/lncRNA
Qing Wang et al	2013	Germany	GSE26886	Affymetrix GPL570	Tissue	mRNA/lncRNA
Hiroki Sasaki et al	2017	Japan	GSE69925	Affymetrix GPL570	Tissue	mRNA/lncRNA
Xiaoyan Ming et al	2017	China	GSE100942	Affymetrix GPL570	Tissue	mRNA/lncRNA
Grace Fu et al	2011	China	GSE33810	Affymetrix GPL570	Tissue	mRNA/lncRNA
Hiroshi Nakagawa et al	2009	USA	GSE17351	Affymetrix GPL570	Tissue	mRNA/lncRNA
Robert J Clifford et al	2011	USA	GSE20347	Affymetrix GPL571	Tissue	mRNA/lncRNA
Howard Yanget al	2015	USA	GSE38129	Affymetrix GPL571	Tissue	mRNA/lncRNA
Wusheng Yanet al	2011	USA	GSE29001	Affymetrix GPL571	Tissue	mRNA/lncRNA
Wusheng Yan et al	2011	USA	GSE33426	Affymetrix GPL571	Tissue	mRNA/lncRNA
Howard Yanget al	2010	USA	GSE23400	Affymetrix GPL96/GPL97	Tissue	mRNA/lncRNA
Yunjuan Bao et al	2012	USA	GSE32424	Illumina GPL10999	Tissue	mRNA/lncRNA
wang liang et al	2013	China	GSE45168	Agilent GPL13497	Tissue	mRNA/lncRNA
Chun-ei Tung et al	2015	China	GSE70409	Phalanx GPL13287	Tissue	mRNA/lncRNA
Wei Wu et al	2013	Canada	GSE45350	Agilent GPL13607	Tissue	mRNA/lncRNA
Jiagen Li et al	2014	China	GSE43732	Agilent GPL16543	Tissue	miRNA
Jing Wen et al	2018	China	GSE114110	Agilent GPL24967	Tissue	miRNA
Rui hua shi et al	2015	China	GSE59973	Agilent GPL16770	Tissue	miRNA
Fen Liu et al	2019	China	GSE112840	Agilent GPL23365	serum	miRNA
Juan Liao et al	2015	China	GSE71043	Agilent GPL18402	blood	miRNA
