# Supplementary material for: Overexpression of UBE2C in esophageal squamous cell carcinoma tissues and molecular analysis
Source: BMC Cancer. 2021 Sep 6;21:996. doi: 10.1186/s12885-021-08634-6 (PMC8422647; doi:10.1186/s12885-021-08634-6)
Supplement: Supplementary file 2 — Additional file 2. [file 12885_2021_8634_MOESM2_ESM.pdf]

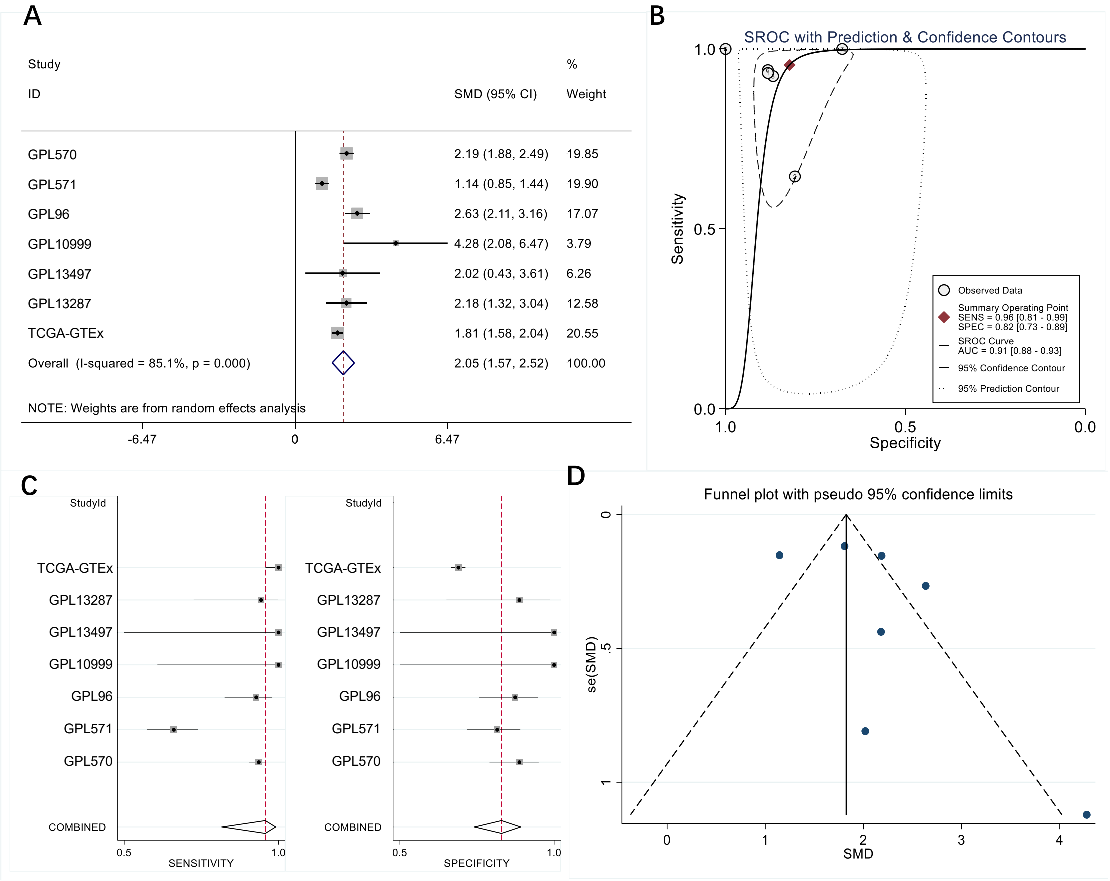

Fig S1. The comprehensive UBE2C expression level in ESCC. a, Forest plot of UBE2C mRNA expression in ESCC based on TCGA-GTEx RNA-seq and tissue microarrays. ESCC vs. normal, random-effects model; b, sROC of UBE2C in ESCC tissues based on TCGA-GTEx RNA-seq and tissue microarrays; c, Sensitivity analysis of TCGA-GTEx RNA-seq and tissue microarrays; d, Funnel plot of TCGA-GTEx RNA-seq and tissue microarrays related to UBE2C.

midas tp fp fn tn, id(accession) ms(0.75) bfor(dss) midas tp fp fn tn, id(accession) ms(0.75) bfor(dss)

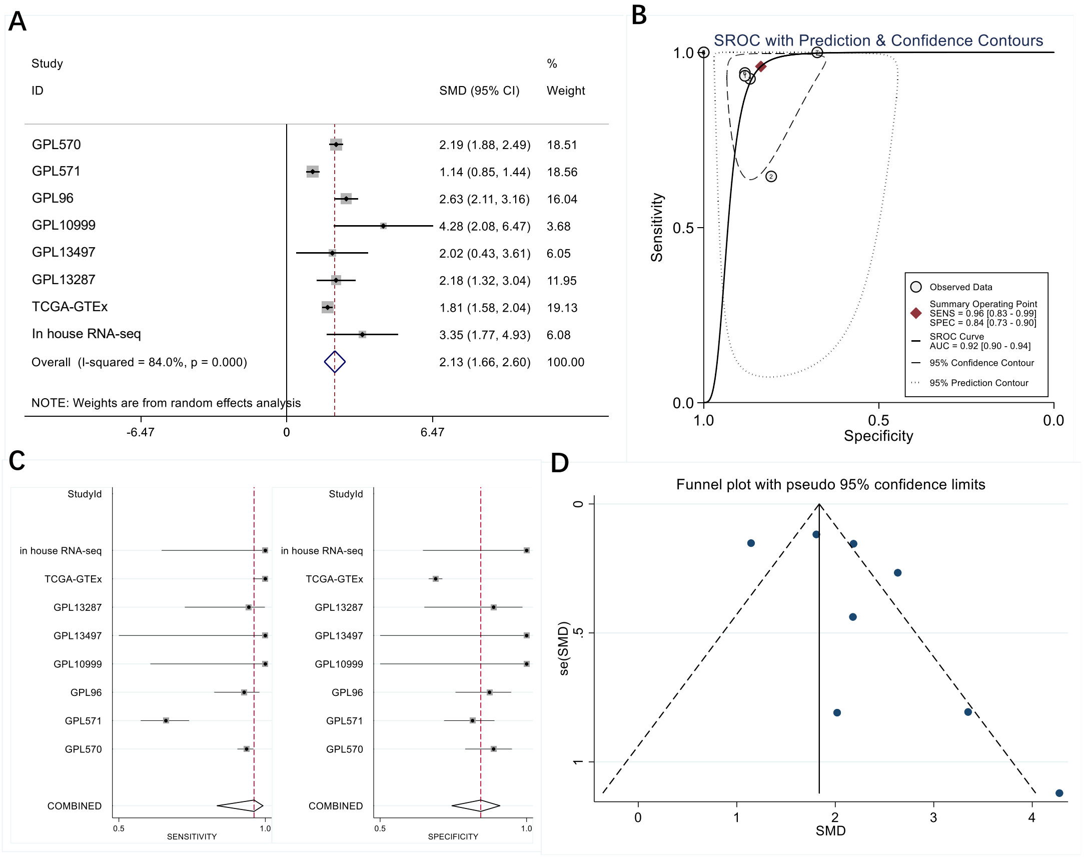

Fig S2. The comprehensive UBE2C expression level in ESCC. a, Forest plot of UBE2C mRNA expression in ESCC based on in house RNA-seq, TCGA-GTEx RNA-seq and tissue microarrays. ESCC vs. normal, random-effects model; b, sROC of UBE2C in ESCC tissues based on in house RNA-seq, TCGA-GTEx RNA-seq and tissue microarrays; c, Sensitivity analysis of in house RNA-seq, TCGA-GTEx RNA-seq and tissue microarrays; d, Funnel plot of in house RNA-seq, TCGA-GTEx RNA-seq and tissue microarrays related to UBE2C.
